# Urinary excretion of homocysteine thiolactone and the risk of acute myocardial infarction in coronary artery disease patients: the WENBIT trial

**DOI:** 10.1111/joim.12834

**Published:** 2018-09-23

**Authors:** K. Borowczyk, J. Piechocka, R. Głowacki, I. Dhar, Ø. Midtun, G. S. Tell, P. M. Ueland, O. Nygård, H. Jakubowski

**Affiliations:** ^1^ Department of Microbiology, Biochemistry and Molecular Genetics Rutgers‐New Jersey Medical School International Center for Public Health Newark NJ USA; ^2^ Department of Environmental Chemistry Faculty of Chemistry University of Łódź Łódź Poland; ^3^ Department of Clinical Science University of Bergen Bergen Norway; ^4^ Bevital AS Bergen Norway; ^5^ Department of Global Public Health and Primary Care University of Bergen Bergen Norway; ^6^ Division of Mental and Physical Health Norwegian Institute of Public Health Bergen Norway; ^7^ Department of Heart Disease Haukeland University Hospital Institute of Medicine University of Bergen Bergen Norway; ^8^ Department of Biochemistry and Biotechnology Poznań University of Life Sciences Poznań Poland

**Keywords:** acute myocardial infarction, atherosclerosis, B‐vitamins, homocysteine thiolactone, paraoxonase

## Abstract

**Objectives:**

No individual homocysteine (Hcy) metabolite has been studied as a risk marker for coronary artery disease (CAD). Our objective was to examine Hcy‐thiolactone, a chemically reactive metabolite generated by methionyl‐tRNA synthetase and cleared by the kidney, as a risk predictor of incident acute myocardial infarction (AMI) in the Western Norway B‐Vitamin Intervention Trial.

**Design:**

Single centre, prospective double‐blind clinical intervention study, randomized in a 2 × 2 factorial design.

**Subjects and methods:**

Patients with suspected CAD (*n* = 2049, 69.8% men; 61.2‐year‐old) were randomized to groups receiving daily (i) folic acid (0.8 mg)/vitamin B_12_ (0.4 mg)/vitamin B_6_ (40 mg); (ii) folic acid/vitamin B_12_; (iii) vitamin B_6_ or (iv) placebo. Urinary Hcy‐thiolactone was quantified at baseline, 12 and 38 months.

**Results:**

Baseline urinary Hcy‐thiolactone/creatinine was significantly associated with plasma tHcy, ApoA1, glomerular filtration rate, potassium and pyridoxal 5′‐phosphate (positively) and with age, hypertension, smoking, urinary creatinine, plasma bilirubin and kynurenine (negatively). During median 4.7‐years, 183 patients (8.9%) suffered an AMI. In Cox regression analysis, Hcy‐thiolactone/creatinine was associated with AMI risk (hazard ratio = 1.58, 95% confidence interval = 1.10–2.26, *P* = 0.012 for trend; adjusted for age, gender, tHcy). This association was confined to patients with pyridoxic acid below median (adjusted HR = 2.72, 95% CI = 1.47–5.03, *P* = 0.0001; *P*_interaction_ = 0.020). B‐vitamin/folate treatments did not affect Hcy‐thiolactone/creatinine and its AMI risk association.

**Conclusions:**

Hcy‐thiolactone/creatinine ratio is a novel AMI risk predictor in patients with suspected CAD, independent of traditional risk factors and tHcy, but modified by vitamin B_6_ catabolism. These findings lend a support to the hypothesis that Hcy‐thiolactone is mechanistically involved in cardiovascular disease.

## Introduction

Clinical studies show that elevated plasma total homocysteine (tHcy), *that is* hyperhomocysteinemia (HHcy), is a risk factor for cardiovascular disease (CVD) and stroke and is a strong predictor of mortality in CVD patients 1, 2. Hcy, an important intermediate in folate and one‐carbon metabolism, is generated from the dietary protein methionine. Genetic and environmental determinants of tHcy are well known 3. Folic acid and B‐vitamin supplementation reduce plasma tHcy by providing cofactors for Hcy‐metabolizing enzymes and have been studied for primary and secondary prevention of CVD outcomes in large‐scale randomized controlled trials (RCTs). Meta‐analyses of eight RCTs involving 37 485 individuals 4 completed by the end of 2009, 24 RTCs involving 57 952 individuals 5 completed by April 2013, and 15 RCTs involving 71 422 participants 6 completed by June 2017 show that tHcy‐lowering by B‐vitamin supplementation has no beneficial effect on risk of myocardial infarction.

A possible explanation for the failure of the tHcy‐lowering trials is that a specific Hcy metabolite that does not respond to folic acid and B‐vitamins is involved. In fact, tHcy is a composite marker that comprises of mostly *S*‐Hcy‐albumin, *S*‐Hcy‐IgG and Hcy‐S‐S‐Cys, with free reduced Hcy itself representing ~1% of the total 1, 2, but does not include other important metabolites, such as Hcy‐thiolactone and product of its reaction with protein (*N*‐Hcy‐protein) 1, 7. Because each of those metabolites can exert a distinct biologic effect 7, a contribution of an individual Hcy metabolite to CVD risk may be confounded or overlooked by using tHcy as a marker 8.

Hcy‐thiolactone is generated in an error‐editing reaction in protein biosynthesis when the noncognate Hcy is selected in place of the cognate methionine by methionyl‐tRNA synthetase 9. Because of its chemical reactivity, Hcy‐thiolactone modifies protein lysine residues in a process called *N*‐homocysteinylation 10, which impairs or alters the protein's structure and function 11, 12, 13.

Because of its exceptionally low pKa of 6.7 12, Hcy‐thiolactone is mostly neutral in the blood and is selectively cleared by the kidney 14. Renal clearance of Hcy‐thiolactone is similar to the clearance of creatinine. Only ~1% of plasma Hcy‐thiolactone filtered in the kidney is reabsorbed in the renal tubules, and >95% is excreted in the urine. This is in contrast to tHcy, 99% of which is reabsorbed and 1% is excreted in the urine. Hcy‐thiolactone concentrations in healthy human subjects are ~100‐fold higher in urine (median 144 nmol L^−1^, range 11–485 nmol L^−1^) 14 than in plasma (median 0.56 nmol L^−1^, range < 0.1–22.6 nmol L^−1^) 15.

Prior studies in cell cultures 10, 16, experimental animals 17 and humans 18 suggest that Hcy‐thiolactone is likely to be mechanistically involved in CVD 12. Because of its efficient renal clearance, urinary Hcy‐thiolactone 14 could provide a sensitive marker of disease. Thus, in the present study, we quantified urinary Hcy‐thiolactone in a large cohort of patients with angiographically confirmed CAD from the Western Norway B‐Vitamin Intervention Trial (WENBIT) 19 and tested the predictive value of urinary Hcy‐thiolactone excretion as a risk marker of acute myocardial infarction (AMI), additionally exploring potential effect modification by B‐vitamin supplementation and status. Preliminary account of this work has been presented at AHA 2015 Scientific Sessions, Orlando, Fl, 7–11 November 2015 ( https://www.ahajournals.org/doi/10.1161/circ.132/suppl_3/19250) 20.

## Methods

### Patients

We analysed existing urine samples from patients with suspected CAD who underwent coronary angiography for stable angina pectoris and participated in the WENBIT study 19. Participant characteristics, blood and urine samples were collected at baseline, 1 year and median 38 months and have been previously described 19. Briefly, the majority of participants had significant coronary stenosis (90%), cardiovascular history/risk factors (60%) and were on medications during the trial, including antiplatelet drugs (92%), acetylsalicylic acid (90.2), statins (88.4%) and β‐blockers (78.2), following baseline angiography. Participants were randomly assigned to groups receiving (i) folic acid (0.8 mg), vitamin B_12_ (cyanocobalamin, 0.4 mg), vitamin B_6_ (pyridoxine, 40 mg); (ii) folic acid and vitamin B_12_; (iii) vitamin B_6_; or (iv) placebo. The study medication (Alpharma Inc, Copenhagen, Denmark) was given as a single capsule, indistinguishable by colour, weight or the ability to dissolve in water.

The present study included 61.9 ± 10.5‐year‐old patients (69.8% male) from baseline (*n* = 2280), 1 year of follow‐up (*n* = 208) and the end of study (*n* = 208). Samples were assayed by investigators blinded to the clinical data to avoid bias. Baseline Hcy‐thiolactone data from 2049 patients for whom urinary creatinine values were available were included in calculations. The study protocol was approved by the Regional ethics committee, by the Norwegian Medicines Agency and the WENBIT Steering Committee.

### Clinical end‐points

The end‐point was incident AMI, which included both fatal and nonfatal events and was defined according to the International Classification of Diseases (ICD) 10th Edition; I21–22. Information on end‐points was obtained from the Cardiovascular Disease in Norway (CVDNOR; https://cvdnor.b.uib.no/) project, which provided information on discharge diagnoses from Norwegian hospitals during 1994–2009, linked to each patient's unique 11‐digit personal number 21.

### Hcy‐thiolactone

Urinary Hcy‐thiolactone was assayed by using solid‐phase extraction (SPE) and high‐performance liquid chromatography (HPLC) with fluorescence detection as previously described.^22^ Briefly, human urine (0.15 mL) was supplemented with sodium phosphate (0.1 mL 0.1 mol L^−1^, pH 7.8) and transferred to Strata C18‐E (55 μm, 50 mg per 1 mL) SPE cartridges (Phenomenex, Torrance, CA, USA), pretreated with 1 mL of 2‐propanol, followed by 0.5 mL 0.2 mol L^−1^ sodium phosphate buffer, pH 7.8. After loading a sample (0.2 mL), the SPE cartridge was washed with 0.25 mL 0.02 mol L^−1^ HCl, 50% methanol solution to remove unwanted material. Hcy‐thiolactone was eluted with 0.15 mL 0.02 mol L^−1^ HCl in 70% acetonitrile–water, and 20 μL of the eluate was loaded on a C18 reversed‐phase HPLC column (Hamilton PRP‐1 column, 150 × 4.6 mm, 5 μm; Energy Way, Reno, NV, USA). The column was eluted at 25 °C with an isocratic solution containing 0.1 mol L^−1^ NaOH, 0.01 mol L^−1^ o‐phthaldialdehyde (MilliporeSigma, St.Louis, MO, USA) and 30% acetonitrile at a flow rate of 1 mL min^−1^. Under these conditions, Hcy‐thiolactone elutes as a sharp peak at 2.1 min, detected and quantified by fluorescence using excitation at 370 nm and emission at 480 nm. An HPLC run‐time for each sample was 3 min. An authentic Hcy‐thiolactone standard (MilliporeSigma) was used for quantification. The intra‐assay and interassay analytical variability is 2.5% and 7%, respectively.

### Other biomarkers

Urinary creatinine 23, plasma pyridoxic acid and pyridoxal 5′‐phosphate (PLP) 19, 24, kynurenine 25, potassium 26, tHcy 23 and ApoA1 were quantified as previously described 19. Data for these biomarkers and for other characteristics of the participants were obtained from the WENBIT database.

### Statistical analysis

Normality of Hcy‐thiolactone distribution was tested with the Shapiro–Wilk's statistic. For normally distributed variables, mean ± standard deviation (SD) was calculated. For non‐normally distributed variables, medians were calculated. An unpaired two‐sided *t*‐test was used for comparisons between two groups of variables with normal distribution. A Mann–Whitney rank sum test was used for comparisons between two groups of non‐normally distributed variables. Associations between Hcy‐thiolactone and other variables were studied by Pearson's correlations and linear regression.

To account for differences in urine dilution amongst participants, urinary Hcy‐thiolactone was normalized to urinary creatinine. Urinary Hcy‐thiolactone/creatinine ratio was log‐transformed because of skewness. Hazard ratios (HR) and 95% confidence intervals (CI) for clinical events were calculated according to tertiles and per 1 SD increment in log‐transformed urinary Hcy‐thiolactone, using Cox regression analysis. Additionally, *P* value for trend across the tertiles was calculated by including the urinary Hcy‐thiolactone in the model as a continuous variable.

The simple model (Model 1) was adjusted for age and sex. Additional covariates for the multivariate model (Model 2) included diabetes (yes or no), hypertension (yes or no), current smoking (yes or no), extent of CAD at angiography (0–3) and left ventricular ejection fraction (LVEF) (%). Event‐free survival was analysed by the Kaplan–Meier method, and log‐rank test was used to estimate differences in survival across Hcy‐thiolactone/creatinine tertiles. Subgroup analyses were performed according to predefined categorical variables or the median value of continuous variable, and effect modification was evaluated by entering interaction product‐term to the Cox model. Moreover, the possibility of unobserved confounding was investigated by applying additional sensitivity analysis to the Cox model 2, according to the recent recommendations for observational studies 27. Statistical software packages SPSS version 15.0 (SPSS Inc., Chicago, IL, USA) and Stats version 10 (StatsCorp LP, College Station, TX, USA) were used. Probability values were two‐sided, and *P* value < 0.05 was considered statistically significant throughout.

## Results

### Baseline Hcy‐thiolactone levels

For the 2280 patients, mean age at baseline was 61.2 years and 29.0% were women. Baseline Hcy‐thiolactone levels varied from 1.3 to 1724 nmol L^−1^ and were significantly higher in men (*n* = 1619) than in women (*n* = 661), with median values 48.6 and 37.6 nmol L^−1^, respectively, *P* = 2 × 10^−6^ (Table 1). After normalizing to creatinine (*n* = 2049), Hcy‐thiolactone/creatinine varied from 0.2 to 128 (nmol L^−1^ per mmol L^−1^, with similar median values for men and women (5.4 vs. 5.8 nmol L^−1^ per mmol L^−1^, *P* = 0.879; Table 1). A total of 14.9% (*n* = 306) of patients reported to be fasting at the time of blood and urine collection. However, the fasting status had no significant effect on mean urinary Hcy‐thiolactone/creatinine values (*P* = 0.727).

**Table 1:** Baseline urinary Hcy‐thiolactone and creatinine levels in CAD patients

Variables	Men (*n* = 1619)		Women (*n* = 661)		*P* value^a^
	Mean ± SD	Median (range)	Mean ± SD	Median (range)	
Hcy‐thiolactone, nmol L^−1^	97.2 ± 46.6	48.6 (1.8–1724)	67.7 ± 100	37.6 (1.3–1019)	2 × 10^−6^
Creatinine, mmol L^−1^	9.9 ± 4.3	9.2 (1.1–31)	7.1 ± 3.7	6.4 (1.0–25.4)	8 × 10^−39^
Hcy‐thiolactone/creatinine, nmol L^−1^ per mmol L^−1^	10.9 ± 17.0	5.4 (0.2–128)	11.1 ± 14.9	5.8 (0.2–125)	0.879
Age, years	61.2 ± 10.4	61 (21–87)	63.1 ± 10.3	63 (28–87)	10^−4^

a *P*‐value for difference between means by gender.

### Determinants of urinary Hcy‐thiolactone excretion

Associations between Hcy‐thiolactone excretion and baseline characteristics are presented in Table 2. In multivariate regression analysis, urinary creatinine, age and eGFR were the strongest determinants of Hcy‐thiolactone excretion (all *P* < 0.0001). Age, urinary creatinine, plasma bilirubin and kynurenine were negatively correlated with Hcy‐thiolactone excretion, whereas tHcy, ApoA1, eGFR and potassium were positively correlated (Table 2). A high Hcy‐thiolactone excretion was associated with higher circulating PLP levels (Table 2), whereas no association was observed with pyridoxal or pyridoxic acid, folate, cobalamin and riboflavin (not shown). There was no association between Hcy‐thiolactone/creatinine and LVEF, body mass index or lipid measures (apolipoprotein B, LDL cholesterol, HDL cholesterol, triglycerides). In addition, mean baseline Hcy‐thiolactone excretion was significantly lower in patients with hypertension and in smokers but was unaffected by diabetes status, previous AMI and CVD status (Table 3), and the extent of CAD (not shown).

**Table 2:** Determinants of urinary Hcy‐thiolactone at baseline*

Pearson correlation			Multivariate regression^a^					
			Model 1		Model 2		Model 3	
Variable	β	*P*	β	*P*	β	*P*	β	*P*
uCreatinine	−0.18	<0.000	−0.20	<0.000	−0.22	<0.000		
Age	−0.13	<0.000	−0.13	<0.000	−0.16	<0.000		
Bilirubin^#^	−0.13	<0.000	−0.09	<0.000	−0.10	<0.000		
Kynurenine	−0.10	<0.000	−0.04	<0.000	−0.06	0.005		
tHcy	0.05	<0.000	0.10	<0.000			0.07	<0.000
eGFR	0.13	<0.000	0.08	0.029			0.18	<0.000
ApoA1	0.09	<0.000	0.08	<0.000			0.11	<0.000
Potassium	0.07	0.001	0.04	0.044			0.11	<0.000
PLP	0.02	0.327	0.03	0.001			0.02	0.336
*n = 2049 ^#^ *n* = 1972			*F* = 18.69; explained variance: *R* ^2^ = 0.09 (*R* = 0.31)		*F* = 41.32; explained variance: *R* ^2^ = 0.08 (*R* = 0.28).		*F* = 19.3; explained variance: *R* ^2^ = 0.04 (*R* = 0.21).	

a anova (Ln[Hcy‐thiolactone/creatinine]).

**Table 3:** Urinary Hcy‐thiolactone levels according to disease status at baseline

Disease (no. of patients)	Ln[HTL/creatinine]		*P* value^a^
	Mean ± SD	Median	
Hypertension (981)	1.71 ± 1.09	1.68	0.008
No hypertension (1063)	1.84 ± 1.16	1.75	
Diabetes (237)	1.83 ± 1.12	1.87	0.408
No diabetes (1812)	1.77 ± 1.13	1.71	
Smokers (1442)	1.83 ± 1.15	1.75	0.001
Non‐smokers (603)	1.64 ± 1.07	1.64	
Previous CVD (1113)	1.74 ± 1.13	1.69	0.150
No previous CVD (936)	1.81 ± 1.13	1.75	
Previous AMI (793)	1.73 ± 1.13	1.68	0.194
No previous AMI (1256)	1.80 ± 1.13	1.74	

a *P* value for difference between means by disease status.

### Urinary Hcy‐thiolactone and the risk of AMI

During a median 4.7‐year follow‐up, 183 (8.9%) patients experienced an AMI. We found that Hcy‐thiolactone/creatinine was significantly associated with the risk of AMI (Table 4). In a model adjusted for age and gender, the HR (95% CI) for AMI was 1.59 (1.11–2.27), when comparing the highest (T3) versus the lowest tertile (T1) of Hcy‐thiolactone/creatinine. Corresponding HRs (95% CIs) were 1.58 (1.11–2.27) in multivariate Cox model 2, and essentially similar after the additional adjustments for medications at discharge (model 3, Table 4). The Hcy‐thiolactone/creatinine and AMI risk association were not affected by adjustment for plasma tHcy (not shown).

**Table 4:** HR (95% CI) for incident AMI according to urinary Hcy‐thiolactone/creatinine

	HR (95% CI) for each tertile of urinary Hcy‐thiolactone/creatinine			*P*_trend_	HR (95% CI) per one SD increment
	T1	T2	T3		
Patients, *n*	683	685	679		
AMI events, *n* (%)	53 (7.8)	58 (8.5)	72 (10.5)		
Model 1^a^	1	1.14 (0.78–1.65)	1.59 (1.11–2.27)	0.011	1.22 (1.05–1.42)
Model 2^b^	1	1.17 (0.81–1.70)	1.58 (1.10–2.26)	0.012	1.22 (1.05–1.41)
Model 3^c^	1	1.16 (0.81–1.69)	1.58 (1.10–2.26)	0.012	1.22 (1.05–1.41)

SD, standard deviation; HR, hazard ratio; CI, confidence interval.

^a^Adjusted for age and gender. ^b^Adjusted for age, gender, hypertension, diabetes, smoking, extent of CAD at angiography and LVEF. ^c^Adjusted for variables in Model 2 plus medications at discharge (including statins, β‐blockers and angiotensin converting enzyme inhibitors and/or angiotensin receptor blocker).

### The Hcy‐thiolactone–AMI risk association is modified by pyridoxic acid

Because Hcy‐thiolactone excretion was associated with PLP, we next examined vitamin B_6_ status as a possible modifiers of the association between Hcy‐thiolactone/creatinine and AMI. We found no effect modification by PLP or pyridoxal, but a significant interaction with a vitamin B6 catabolite, pyridoxic acid (Table 5). Notably, a positive association between Hcy‐thiolactone/creatinine and the risk of AMI was confined to patients with low (below median) pyridoxic acid (HR: 2.72 [1.47–5.03], *P* = 0.001), whereas there was no risk association amongst patients with high pyridoxic acid (above median) (*P*_interaction_ = 0.020).

**Table 5:** Pyridoxic acid modifies the risk of AMI due to urine Hcy‐thiolactone/creatinine in CAD patients

Patients	Hazard ratio (95% CI)^a^		*P*_interaction_
	T3 vs. T1 of Hcy‐thiolactone/creatinine	*P* value	
All (*n* = 2049)	1.23 (1.025–1.474)	0.026	0.020
Pyridoxic acid < median	2.72 (1.47–5.03)	0.001	
Pyridoxic acid > median	0.99 (0.62–1.56)	0.95	
No diabetes and hypertension (*n* = 508)	3.11 (1.21–7.96)	0.018	
Pyridoxic acid < median	4.79 (1.01–22.65)	0.048	
Pyridoxic acid > median	2.13 (0.63–7.17)	0.223	

a Adjusted for age, gender, diabetes, hypertension, smoking, tHcy, extent of CAD at angiography and LVEF.

The interaction with pyridoxic acid was further plotted in a VisGAM surface plot (Fig. S1). At low pyridoxic acid, the risk of AMI increased linearly from 9.5% to 18% with the increase in Hcy‐thiolactone/creatinine ratio, whereas no relationship was observed at high pyridoxic acid. Kaplan–Meier analysis shows that patients with low pyridoxic acid have a significantly better survival free of AMI (Fig. S2).

In a subgroup analysis, we found that the positive association between Hcy‐thiolactone/creatinine and the risk of AMI was stronger in patients without diabetes and hypertension at baseline, *that is*, metabolically healthy (Table 5). Similar to the whole cohort, the Hcy‐thiolactone–AMI risk association was modified by plasma pyridoxic acid and was confined to patients with low (below median) pyridoxic acid (HR: 4.79 [1.01–22.65], *P* = 0.048), whereas there was no risk association amongst patients with high (above median) plasma pyridoxic acid (Table 5). We observed no interaction with smoking, age or gender (data not shown).

### Supplementation with folic acid, vitamin B_12_ and/or vitamin B_6_ does not affect Hcy‐thiolactone levels and Hcy‐thiolactone/creatinine ratios during follow‐up

A previous study of this cohort 19 found that mean serum folate increased sevenfold, and cobalamin increased by 65% in the groups receiving folic acid + vitamin B_12_. Mean plasma pyridoxal 5′‐phosphate increased ninefold in the groups receiving vitamin B_6_. At the same time, plasma tHcy decreased by 30% after 12 months and 26% after 38 months in the groups receiving folic acid + vitamin B_12_ (10.8 ± 4.5 μmol L^−1^ at baseline to 7.6 ± 2.2 μmol L^−1^, *P* < 0.001)^19^. Plasma tHcy was unaltered in the groups receiving vitamin B_6_ alone or placebo.

We found that after 12 and 38 months of supplementation with any combination of folic acid and B‐vitamins, urinary Hcy‐thiolactone levels were unaltered relative to placebo at both time‐points. Hcy‐thiolactone normalized to creatinine also remained unaltered after supplementation (Table 6).

**Table 6:** Hcy‐thiolactone/creatinine according to folic acid, B‐vitamin supplementation status

Treatment group (*n* = 52)	Hcy‐thiolactone/creatinine, nmol L^−1^ per mmol L^−1^				*P* a versus placebo	
	12 months		38 months			
	Mean ± SD	Median (range)	Mean ± SD	Median (range)	12 months	38 months
(i) FA+B_12_+B_6_	14.0 ± 21.4	6.3 (0.5–113)	8.9 ± 14.5	4.3 (0.5–100)	0.842	0.907
(ii) FA+B_12_	17.0 ± 21.5	6.8 (0.4–87)	10.6 ± 11.7	5.9 (0.2–63)	0.842	0.346
(iii) B_6_	11.8 ± 16.9	5.7 (0.6–92)	11.2 ± 18.8	5.6 (0.6–107)	0.906	0.350
(iv) Placebo	11.1 ± 15.8	6.0 (0.8–97)	8.6 ± 14.2	5.0 (0.7–94)		

a *P* value for difference between means.

### Sensitivity analysis

Application of E‐value formula to the multivariate Cox model 2 (Table 4) revealed high sensitivity of the observed association between urinary Hcy‐thiolactone/creatinine and the risk of AMI, with an E‐value of 2.53 for the total estimate and 1.43 each for lower reported CI.

### Coefficients of reliability

To determine the reliability of a single measurement of Hcy‐thiolactone, we quantified Hcy‐thiolactone in randomly selected 100 patients at three time‐points in the WENBIT trial: at baseline, at 12 and 38 months. The median and mean Hcy‐thiolactone concentrations at baseline were 43.8 and 88.3 nmol L^−1^, respectively. The between‐person SD was 133.8 nmol L^−1^ and CV was 151%. The mean of three measurements of Hcy‐thiolactone over a 38‐month time interval was 56.4 nmol L^−1^, and the within‐person SD was 65.5 nmol L^−1^, and CV was 116%. The reliability coefficient (between‐person variance as a proportion of the total variance, calculated according to: reliability coefficient = 1/[1 + (within‐person SD/between‐person SD)^2^] for a single measurement of Hcy‐thiolactone was 0.81 and compared favourably with values of reliability coefficients of 0.88 for tHcy, 0.85 for total cholesterol and 0.74 for systolic blood pressure 28.

## Discussion

Prior studies on the relationships between HHcy and CVD outcomes relied on quantification of ‘total Hcy’, a composite marker 1, as an indicator of HHcy. No individual Hcy metabolite has been previously studied in large‐scale observational investigation in the context of CVD. In the present study, we examined a relationship between Hcy‐thiolactone, a well‐defined, chemically reactive metabolite of Hcy 9 and a risk of AMI. We found that (i) urinary Hcy‐thiolactone was only weakly associated with plasma tHcy levels but varied considerably between individuals; (ii) urinary Hcy‐thiolactone, normalized to creatinine, is a risk marker of future AMI events in CAD patients; (iii) adjustment for tHcy did not influence the Hcy‐thiolactone AMI risk association; (iv) the positive relationship between Hcy‐thiolactone/creatinine and the AMI risk is modified by plasma pyridoxic acid, the catabolite of vitamin B_6_; (v) folic acid and B‐vitamin supplementation, which has no effect on AMI events, also does not have any significant effect on Hcy‐thiolactone levels.

The baseline urinary Hcy‐thiolactone exhibited an exceptionally high interindividual variation (1326‐fold; from 1.3 to 1724 nmol L^−1^, Table 1), compared to a much more limited interindividual variation of tHcy or other metabolites in the WENBIT cohort. However, higher interindividual variation of Hcy‐thiolactone relative to tHcy has also been observed in other studies. For example, in CBS‐deficient patients on a tHcy‐lowering therapy (*n* = 14), plasma Hcy‐thiolactone varies 1000‐fold between individuals whilst plasma tHcy varies only 6.2‐fold 29. In a cohort (*n* = 60) of healthy subjects, interindividual variation of plasma Hcy‐thiolactone and tHcy is >97‐fold and 6.7‐fold, respectively 15. Interindividual variation of urinary and plasma Hcy‐thiolactone is 44‐fold and >226‐fold, respectively, whilst the corresponding variation of tHcy is 13‐fold and 5.3‐fold, respectively 14. However, why Hcy‐thiolactone varies so much between individuals is not clear.

Although urinary biomarkers can provide a noninvasive tool for diagnosis and monitoring of human disease, only a few biomarkers of CVD risk have been identified in the human urine. These include inflammation markers that are elevated in the urine of diabetes mellitus patients 30 and urinary betaine that is an independent predictor of the development of diabetes 23. In addition, urinary collagen peptide patterns, identified by capillary electrophoresis–mass spectrometry, can distinguish patients with CVD from healthy controls 31. With the exception of microalbuminuria, an established CVD risk marker 32, urinary biomarkers have not been extensively studied in atherosclerosis. More recently, the urinary kynurenine/tryptophan ratio was found to be strongly associated with adverse long‐term prognosis, including major coronary events, AMI and all‐cause mortality in patients with CAD 25. The present study adds Hcy‐thiolactone to a short list of known urinary biomarkers of the CVD risk.

**Figure 1:**
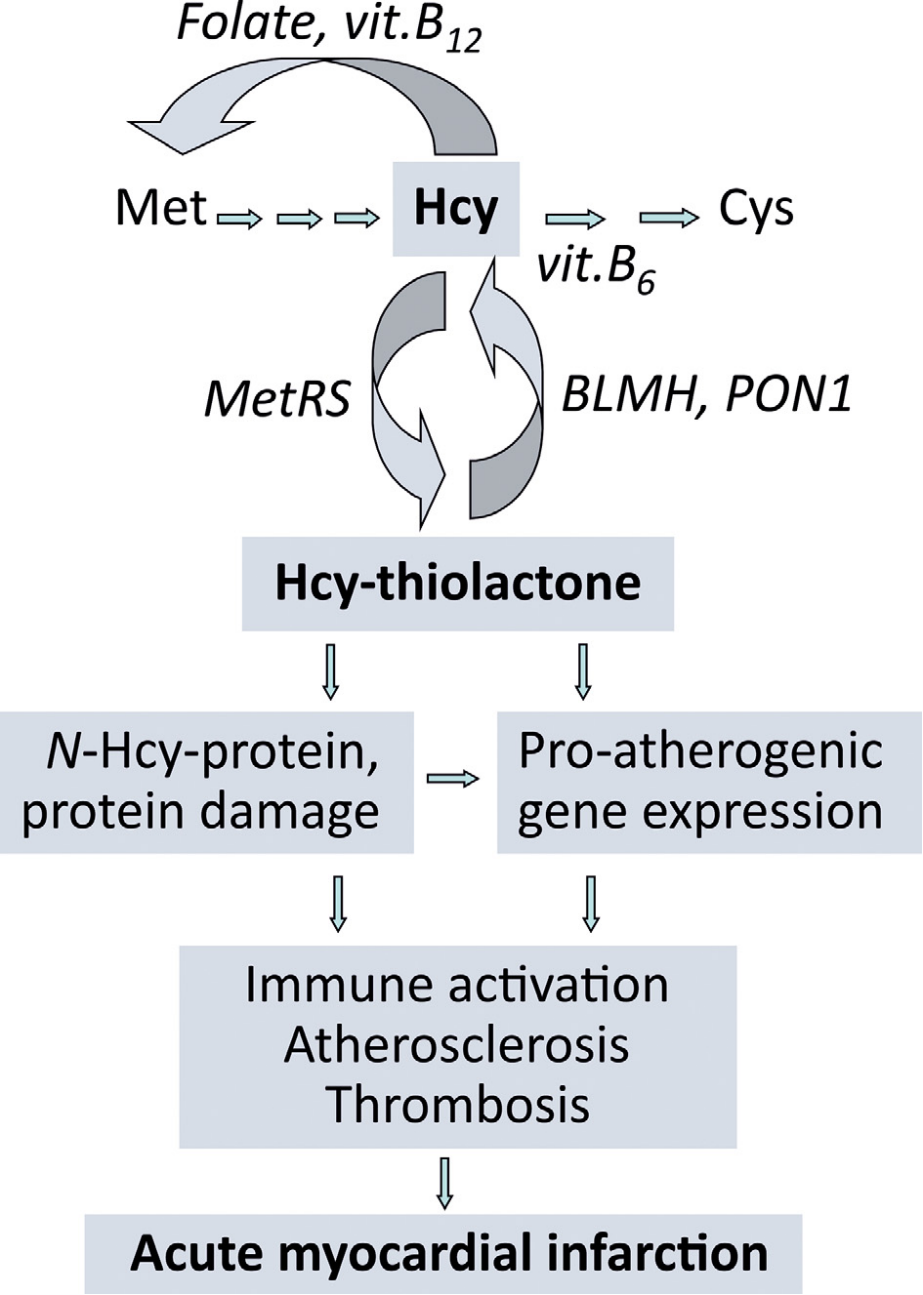
The Hcy‐thiolactone hypothesis of CVD.

The thioester chemistry of Hcy‐thiolactone underlies its ability to form stable iso‐peptide bonds with protein lysine residues in a process called *N*‐homocysteinylation and thus to impair or alter the protein's biological function, a hallmark of many diseases, including CVD. The discoveries of Hcy‐thiolactone in human cells 10 and body 15, 29 and of its reactions with proteins *in vitro* 11 and *in vivo* in human cells 10 and tissues 18 led to the Hcy‐thiolactone hypothesis (Fig. 1), which states that metabolic conversion of Hcy to Hcy‐thiolactone and concomitant protein *N*‐homocysteinylation underlie the involvement of HHcy in CVD 7, 10. Findings of the present study showing that urinary Hcy‐thiolactone predicts future AMI events in CAD patients support this hypothesis.

That Hcy‐thiolactone can be involved in CVD is also supported by previous animal and human studies. For example, chronic infusions of baboons with Hcy‐thiolactone produce patchy desquamation of vascular endothelium, appearance of circulating endothelial cells and typical arteriosclerotic lesions and arterial thrombosis 33. Further, mice deficient in their ability to hydrolyse Hcy‐thiolactone due to inactivation of the *Pon1* or *Blmh* gene are significantly more susceptible to Hcy‐thiolactone‐induced seizures than wild‐type animals 34, 35. Further, Hcy‐thiolactone is cytotoxic 36 and induces pro‐atherogenic changes in gene expression in human vascular endothelial cells 16. The present findings that urinary Hcy‐thiolactone is a predictor of AMI events lend additional support to the theory that Hcy‐thiolactone is involved in the pathology of CVD (Fig. 1).

**Figure 2:**
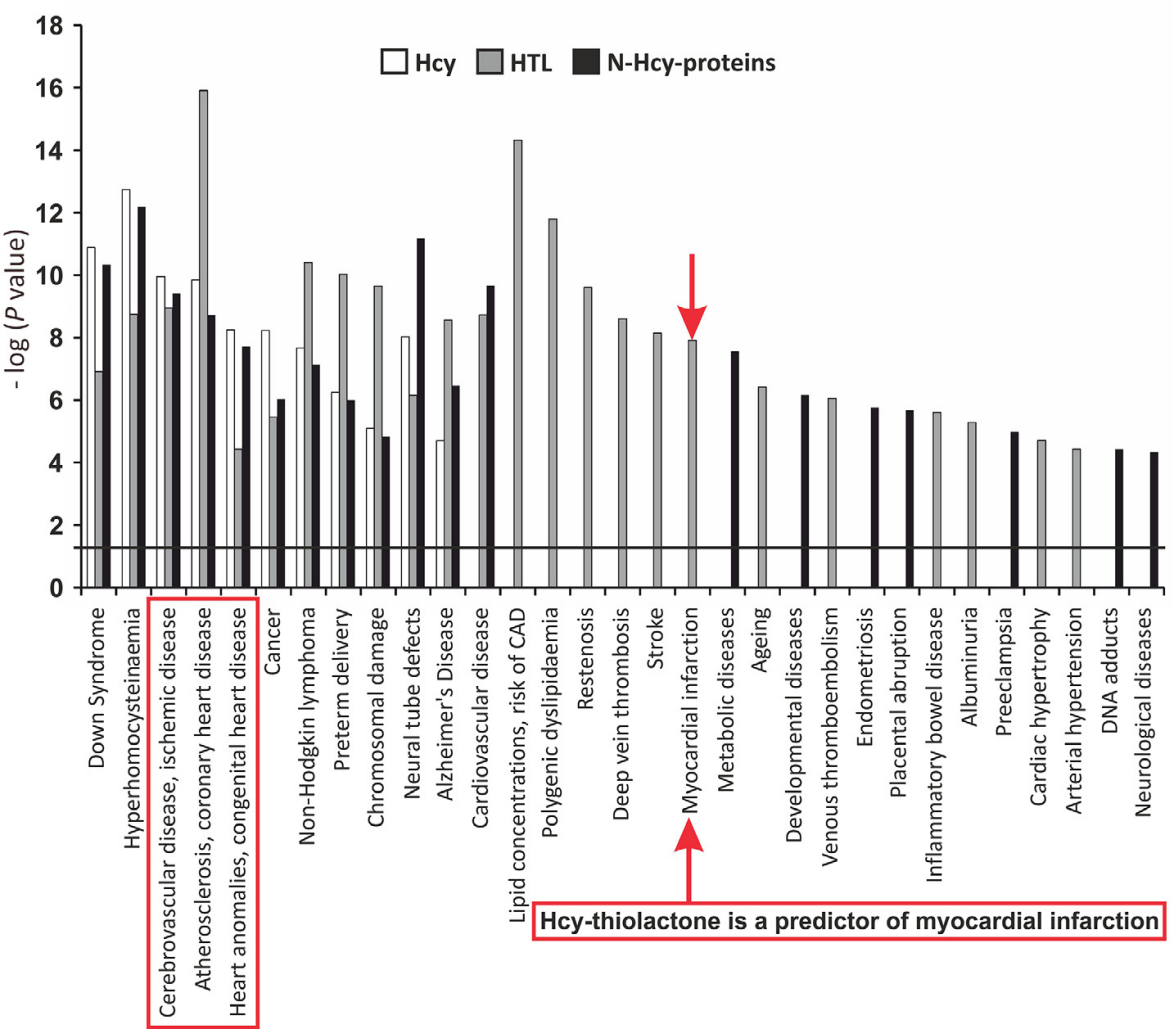
Diseases associated with Hcy‐thiolactone, *N*‐Hcy‐protein and Hcy deduced from effects of these metabolites on gene expression in human vascular endothelial cells. Modified from Ref. 16.

The present finding that urinary Hcy‐thiolactone/creatinine predicts AMI is consistent with bioinformatic analyses of changes in gene expression induced in human vascular endothelial cells by treatments with Hcy‐thiolactone, *N*‐Hcy‐protein or Hcy, which show that myocardial infarction is strongly associated with Hcy‐thiolactone (Fig. 2), but not with Hcy 16. Further, the Gurda *et al*. study also shows that CVD is associated with Hcy‐thiolactone and *N*‐Hcy‐protein, but not with Hcy. However, all three metabolites are associated with cerebrovascular disease, atherosclerosis and coronary heart disease (Fig. 2) 16. Taken together, these data suggest a prominent role of Hcy‐thiolactone in CVD and show that each of those metabolites can affect different aspects of the disease.

Notably, *N*‐homocysteinylation increases protein's susceptibility to oxidative damage and causes formation of toxic protein aggregates 11, 12. Further, the plausibility that protein *N*‐homocysteinylation can play a role in atherothrombosis is suggested by the accumulation of pro‐thrombotic *N*‐Hcy‐fibrinogen in CBS‐deficient patients 18. In cardiac surgery patients, *N*‐Hcy‐protein accumulates in myocardium and aorta 37. Animal studies show that *N*‐Hcy‐protein accumulates within atherosclerotic lesions in aortas of *ApoE* ^*−/−*^ mice fed with a normal chow diet and that the accumulation increases in the *ApoE* ^*−/−*^ animals fed with a HHcy diet‐37. Other data show that *N*‐homocysteinylation impairs collagen cross‐linking in various organs, including the heart, of *Cbs* ^−/−^ mice, thus providing an explanation for connective tissue deficiencies prevalent in HHcy humans 13. In small, case–control studies, elevated plasma Hcy‐thiolactone and *N*‐Hcy‐protein are associated with vascular damage in diabetes 38 and increased CAD risk 39, respectively.

In humans, the accumulation of *N‐*Hcy‐protein triggers an auto‐immune response (Fig. 1), which is associated with CAD 40 and stroke 41. Anti‐*N*‐Hcy‐protein IgG auto‐antibodies 41 and *N*‐Hcy‐protein levels 42 vary considerably amongst individuals and are strongly correlated with plasma tHcy, but not with Cys or Met 41. Such correlations suggest a mechanistic link between these Hcy‐related species: elevation of Hcy levels leads to inadvertent elevation of Hcy‐thiolactone 10, 29, which mediates protein *N*‐homocysteinylation and thus the generation of neo‐self *N*ε‐HcyLys antigens. Raising levels of these antigens trigger an auto‐immune response. Auto‐antibodies recognizing the *N*ε‐HcyLys epitope can react with any *N*‐Hcy‐protein 41 in any tissue, thus contributing to deleterious effects of HHcy on many organs, including the heart. If the neo‐self *N*ε‐HcyLys epitopes were present on endothelial cell membrane proteins, anti‐*N*‐Hcy‐protein auto‐antibodies would form antigen‐antibody complexes on the surface of the vascular vessel. Endothelial cells coated with anti‐*N*‐Hcy‐protein auto‐antibodies would be taken up by the macrophage *via* the Fc receptor, resulting in an injury to the vascular intima. If the *N*‐Hcy‐proteins were present chronically, repeating attempts to repair the damaged vascular wall would lead to a lesion, a hallmark of atherosclerosis.

The involvement of anti‐*N*‐Hcy‐protein auto‐antibodies in CAD (Fig. 1) is supported by findings that lowering tHcy by folic acid (for 3 and 6 months) lowers anti‐*N*‐Hcy‐protein autoantibody levels in healthy individuals but not in CAD patients 43. Thus, primary tHcy‐lowering is beneficial because it reduces an auto‐immune response. In contrast, secondary tHcy‐lowering is not beneficial because it does not reduce anti‐*N*‐Hcy‐protein autoantibody levels nor inflammation (CRP) 43, as also shown by analysis of CRP and other inflammatory markers (IL‐6, sCD40L, neopterin) in a small subset of WENBIT patients 24. Thus, the failure of B‐vitamin treatments to reduce inflammation could account for the lack of efficacy of tHcy‐lowering trials to reduce cardiovascular events in patients with CVD. Our findings that supplementation with B‐vitamins does not significantly affect Hcy‐thiolactone could provide an additional explanation accounting for the lack of efficacy of tHcy‐lowering trials: the B‐vitamin intervention is ineffective because it has no influence on Hcy‐related pro‐atherogenic factors, such as Hcy‐thiolactone and anti‐*N*‐Hcy‐protein auto‐antibodies. Thus, targeting Hcy‐thiolactone might be a more effective tactic for prevention of HHcy‐related CVD.

That the association between Hcy‐thiolactone and the risk of AMI is modified by pyridoxic acid and is observed at low (below median) pyridoxic acid (Table 4) suggests that vitamin B_6_ catabolism could contribute to the risk. However, the mechanism underlying the interaction between Hcy‐thiolactone and pyridoxic acid in the risk of AMI remains to be investigated in future studies.

Our present finding that Hcy‐thiolactone predicts AMI underscores the need for identification of factors affecting Hcy‐thiolactone levels in humans. In the present study, we identified five positive (tHcy, ApoA1, eGFR, potassium and PLP) and four negative (age, creatinine, bilirubin and kynurenine) determinants of baseline Hcy‐thiolactone excretion (Table 2). Hypertension and smoking were also negatively associated with baseline Hcy‐thiolactone (Table 3). Interestingly, although men had significantly higher baseline urinary Hcy‐thiolactone than women, this reflected higher metabolic rate in men than in women, and there was no significant effect of gender on Hcy‐thiolactone after normalizing to creatinine (Table 1).

Positive association between Hcy‐thiolactone and tHcy, also observed previously in a smaller studies 14, 44, is consistent with Hcy being a substrate for the synthesis of Hcy‐thiolactone 9, 10. The negative association between Hcy‐thiolactone and hypertension may be mediated by potassium, whose low intake and plasma levels are an established risk factor for hypertension.^26^ Negative associations of Hcy‐thiolactone with bilirubin and kynurenine suggest that Hcy‐thiolactone can be involved in catabolism of haemoglobin and tryptophan, respectively. However, the molecular basis for these associations is unclear and remains to be investigated. As these determinants explain only 4–9% of the Hcy‐thiolactone excretion variance (Table 2), other determinants remain to be discovered. Likely candidates (Fig. 1) include methionyl‐tRNA synthetase (MARS), responsible for the synthesis of Hcy‐thiolactone 9, 10 and HTases such as PON1, BLMH and BPHL 45, 46, 47, 48, which are responsible for Hcy‐thiolactone hydrolysis. Indeed, recent studies found that PON1 Q192R genotype and activity affect Hcy‐thiolactone levels in humans 49 and that among CAD patients undergoing percutaneous coronary intervention those with low serum HTase activity show significantly higher all‐cause mortality than patients with high HTase activity 50.

### Strengths and limitations

The present study is the first to evaluate Hcy‐thiolactone, a marker of protein‐related Hcy metabolism, for prognostication of CAD patients. With over 2000 participants, this is also the largest study that evaluated Hcy‐thiolactone as a predictor of future AMI events in humans, allowing assessment of effect estimates in subgroups. Other important strengths include the prospective design, and extensive information regarding baseline clinical characteristics. The information on incident AMI outcomes during the follow‐up was ascertained using a patient administrative and a population‐based registry. Moreover, the high E‐value for total HR as well as lower CI suggests that the current observed findings are robust to the presence of an unmeasured cofounding and, therefore are likely to be reproducible in other populations.

Urine samples were stored at room temperature for a few hours before freezing at −80°C. This, however, is unlikely to introduce bias, because Hcy‐thiolactone in urine is stable for at least 24 h under these conditions 14. Furthermore, the majority of patients did not fast before sampling. Since Hcy‐thiolactone/creatinine values did not differ significantly between the fasting and nonfasting groups, the fasting status is unlikely to affect our conclusions. We mainly studied white, elderly patients with CAD, and our results may not be applicable to subjects in other age group and ethnic background or to general healthy population. Notably, however, urinary Hcy‐thiolactone was a strong predictor even in the subgroup with presumably lower cardiovascular risk (without diabetes and hypertension).

## Conclusions

We and other investigators have linked a chemically reactive Hcy metabolite, Hcy‐thiolactone, to CVD by elucidating in laboratory studies possible molecular mechanisms underlying Hcy‐thiolactone involvement, confirmed those mechanisms in studies in humans and experimental animals and provided evidence from pathology studies in human CVD patients and animal models. Predictions from those initial studies are confirmed in the present study of a large cohort of patients with suspected stable angina pectoris, which suggests Hcy‐thiolactone/creatinine ratio to be a new strong risk predictor of incident AMI, particularly in subgroups with pyridoxic acid levels below the median. Our findings support a hypothesis that Hcy‐thiolactone is mechanistically involved in CVD and motivate further research to elucidate the role of Hcy‐thiolactone in atherosclerotic cardiovascular disease development. These findings also suggest that targeting Hcy‐thiolactone might be a more effective tactic for prevention of HHcy‐related disease.

## Conflict of interest statement

None.

## Funding sources

This study was supported in part by grants from the National Science Centre, Poland (2011/02/A/NZ1/00010, 2012/07/B/NZ7/01178, 2013/09/B/NZ5/02794, 2013/11/B/NZ1/00091, 2016/23/B/NZ5/00573), and the American Heart Association (0855919D, 12GRNT9420014).

## Author contributions

KB, JP and RG quantified Hcy‐thiolactone levels; ID and ON were responsible for securing the data quality for variables in the WENBIT database; ØM and PMU were responsible for B6 vitaminers; GST collected the follow‐up data on incident myocardial infarction; ON, ID and HJ analysed the data; HJ designed the research, wrote the manuscript and had primary responsibility for the final content; and all authors read and approved the final manuscript.

## Supporting information


**Figure S1.** A surface plot depicting increase in risk of AMI by quintiles of log‐transformed Hcy‐thiolactone/creatinine and pyridoxic acid. The prevalence of AMI events in patients without vitamin B_6_ supplementation was analyzed (*n* = 1411).
**Figure S2.** Kaplan‐Meier analysis of AMI events according to tertiles of Hcy‐thiolactone/creatinine ratios.
