# Supplementary material for: Urinary excretion of homocysteine thiolactone and the risk of acute myocardial infarction in coronary artery disease patients: the WENBIT trial
Source: J Intern Med. 2018 Sep 23;285(2):232–44. doi: 10.1111/joim.12834 (PMC6378604; doi:10.1111/joim.12834)
Supplement: Supplementary file 1 — Figure S1. A surface plot depicting increase in risk of AMI by quintiles of log‐transformed Hcy‐thiolactone/creatinine and pyridoxic acid. The prevalence of AMI events in patients without vitamin B6 supplementation was analyzed (n = 1411). Figure S2. Kaplan‐Meier analysis of AMI events according to tertiles of Hcy‐thiolactone/creatinine ratios. [file JOIM-285-232-s001.docx]

**Supplemental File, online Data Supplement.**

**Figures: 2**


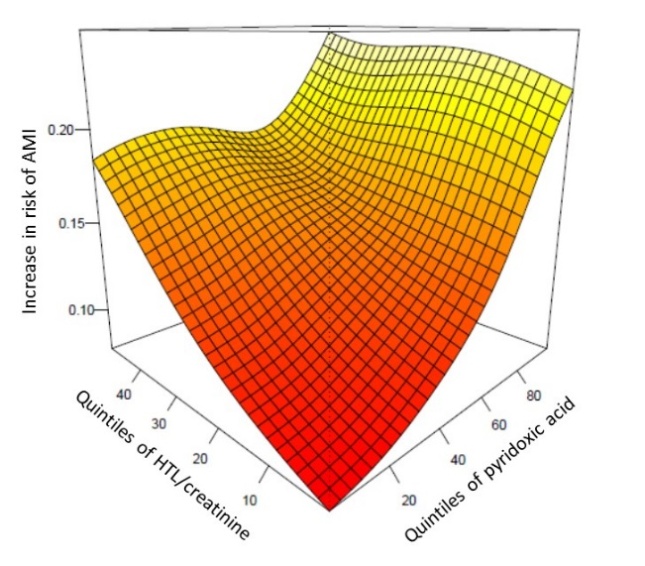


**Figure S1.** A surface plot depicting increase in risk of AMI by quintiles of log-transformed Hcy-thiolactone/creatinine and pyridoxic acid. The prevalence of AMI events in patients without vitamin B_6_ supplementation was analyzed (n=1,411).


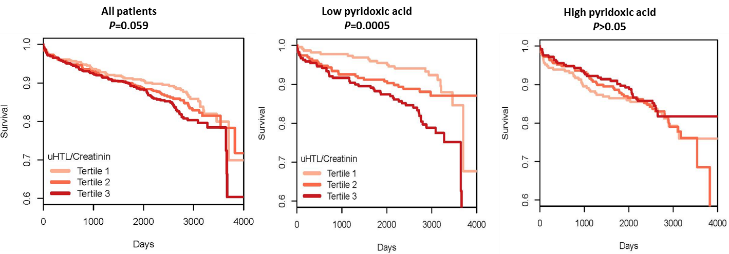


**Figure S2.** Kaplan-Meier analysis of AMI events according to tertiles of Hcy-thiolactone/creatinine ratios.
