# Supplementary figures and images for: Urinary excretion of homocysteine thiolactone and the risk of acute myocardial infarction in coronary artery disease patients: the WENBIT trial
Source: J Intern Med. 2018 Sep 23;285(2):232–44. doi: 10.1111/joim.12834 (PMC6378604; doi:10.1111/joim.12834)

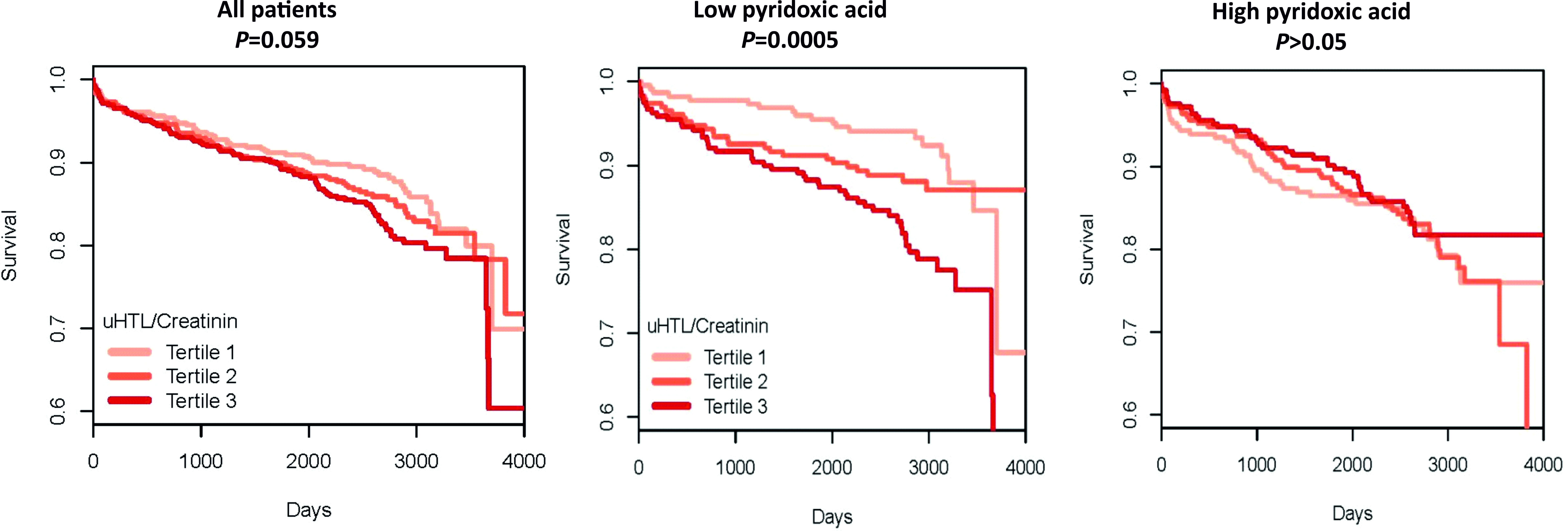

Supplement: Supplementary file 3 [file JOIM-285-232-s003.jpg]
